# Lipid metabolism in tumor-infiltrating T cells: mechanisms and applications

**DOI:** 10.1093/lifemeta/loac038

**Published:** 2022-12-15

**Authors:** Xin-Yu Ke, Miaowen Zou, Chenqi Xu

**Affiliations:** State Key Laboratory of Molecular Biology, Shanghai Science Research Center, CAS Center for Excellence in Molecular Cell Science, Shanghai Institute of Biochemistry and Cell Biology, Chinese Academy of Sciences, Shanghai 200031, China; State Key Laboratory of Molecular Biology, Shanghai Science Research Center, CAS Center for Excellence in Molecular Cell Science, Shanghai Institute of Biochemistry and Cell Biology, Chinese Academy of Sciences, Shanghai 200031, China; State Key Laboratory of Molecular Biology, Shanghai Science Research Center, CAS Center for Excellence in Molecular Cell Science, Shanghai Institute of Biochemistry and Cell Biology, Chinese Academy of Sciences, Shanghai 200031, China

**Keywords:** T cells, lipid metabolism, fatty acid metabolism, cholesterol metabolism, tumor microenvironment, cancer immunotherapies

## Abstract

As an essential part of adaptive immunity, T cells coordinate the immune responses against pathogens and cancer cells. Lipid metabolism has emerged as a key regulator for the activation, differentiation, and effector functions of T cells. Therefore, uncovering the molecular mechanisms by which lipid metabolism dictates T cell biology is of vital importance. The tumor microenvironment is a hostile milieu, i.e. often characterized by nutrient restriction. In this environment, various cells, such as T cells and cancer cells, reprogram their metabolism, including their lipid metabolism, to meet their energy and functional needs. Here, we review the participation of fatty acid and cholesterol metabolism homeostasis in orchestrating T cell biology. We demonstrate how the tumor microenvironment reshapes the lipid metabolism in T cells. Importantly, we highlight the current cancer therapeutic interventions that target fatty acid and cholesterol metabolism of T cells. By offering a holistic understanding of how lipid metabolic adaption by T cells facilitates their immunosurveillance in the tumor microenvironment, we believe this review and the future studies might inspire the next-generation immunotherapies.

## Introduction

T cells are vital components of the adaptive immune response that can discriminate between ‘self’ and ‘non-self’ antigens and build a barrier to defend against pathogens and cancers. Functional T cells can be classified into several types. Upon activation, naïve CD4^+^ T cells can proliferate and differentiate into different subsets, including helper T (Th) cells and regulatory T (Treg) cells, with a more fine classification of Th cells into Th1, Th2, Th17, and follicular helper T (Tfh) cells [1]. In addition, naïve CD8^+^ T cells can differentiate into a population of effector T (Teff) cells, followed by the release of enzymes and toxins and executing killing functions against infections and cancers, while the population of CD8^+^ memory T (Tmem) cells have the ability to maintain long-term rapid response to the reintroduction of relevant pathogens after an infection has been eliminated. Elucidating the fate mediators that maintain T cell function will establish a better understanding of T cell biology and thus provide opportunities for more efficient development of therapeutic approaches to modulate the immune system.

Mounting evidence suggests that metabolic flexibility is actively involved in the differentiation and function of T cells. Normally, to maintain their active proliferative capacity, T cells sustain high levels of energy expenditure, which is mostly provided by glycolysis. But under certain circumstances, lipids, which are essential nutrients, play a vital role in meeting energy demands in T cells [2]. Based on their structures, lipids can be divided into three categories, including simple lipids, compound lipids, and derived lipids, with fatty acids and cholesterol belonging to the third category and contributing to a large proportion of the total lipid pool [3]. Up to now, the biological functions of lipids have been well identified, including comprising the main components of cellular and plasma membranes, providing a source of cellular energy, and acting as mediators of various signaling pathways. Hence, the homeostasis and normal functions of lipids should be carefully maintained by T cells. Lipid homeostasis is regulated by several processes, including *de novo* synthesis, uptake and catabolism, and so far. The role of lipid homeostasis in T cell fate determination has been extensively studied in diverse settings. For instance, fatty acids have been suggested to be the key energy resource for naïve T cells and Tmem [4, 5]. It is also shown that CD4^+^ T cells tend to enhance fatty acid synthesis during Th17 differentiation conditions [6]. All these suggest important roles of lipid metabolism in T cells.

Meanwhile, within the tumor microenvironment (TME), cancer cells also need to utilize lipids [7]. Lipids not only serve as an alternative energy source, but also provide the materials of biological membranes, both of which are essential for cancer cell proliferation. Furthermore, cell–cell communication within the TME is also regulated by the accumulation of signaling lipids, including sphingolipids, fatty acids, eicosanoids, and phosphoinositides, which might further facilitate tumorigenesis [8, 9]. Though lipid metabolic reprogramming in cancer cells and T cells are both emerging topics of keen interest, the connections between lipid metabolism, T cells and the TME have not been well established, and our current understanding of T cell metabolism in the TME remains poorly defined. Therefore, in this review, we intend to highlight how lipid metabolism, especially fatty acid metabolism and cholesterol metabolism, is modulated in T cell physiology. We discuss the role of lipid metabolism in T cells in the context of cancer, especially how metabolism affects their ability to facilitate or inhibit tumor progression. We also summarize the current therapeutic interventions targeting T cell lipid metabolism. Together, we hope the review will broaden the knowledge regarding T cell metabolism to inspire future studies and the development of additional immunotherapies to cure cancer or other diseases.

## Fatty acid homeostasis

Fatty acids are composed of saturated or unsaturated hydrocarbon chains that terminate with carboxylic acid groups. As important components of lipids, fatty acids and their derivates are actively involved in fuel replenishment, signal transduction, cell membrane formation, and energy storage. Fatty acid metabolism consists of anabolism, catabolism, and storage, and helps to maintain the cellular lipid content and homeostasis. In this section, we will cover the functional involvement of fatty acid metabolism in T cells.

### Fatty acid *de novo* synthesis

Typically, *de novo* fatty acid synthesis requires acetyl-CoA as the starting material, followed by its conversion into malonyl-CoA by the enzyme acetyl-CoA carboxylase 1 (ACC1). Malonyl-CoA and acetyl-CoA are further synthesized into long-chain fatty acids (LCFAs), a process catalyzed by fatty acid synthase (FASN). Therefore, ACC1 and FASN are two key enzymes involved in fatty acid *de novo* synthesis. In this regard, several studies have explored the contribution of lipogenic enzymes to the activation and preservation of T cells. Upon T cell activation, higher expression of ACC1 promotes the differentiation of naïve CD4^+^ T cells into Th1 or Th2 effector cells, while a low level of ACC1 profoundly enhances Tmem cell formation. ACC1 also facilitates the development of pathogenic Th2 cell populations by constraining interleukin (IL)-3 or IL-5 production, causing allergic eosinophilic airway inflammation [10]. Similarly, manipulating *ACC1* expression and thus the subsequent *de novo* fatty acid synthesis pathway has been reported to alter the balance between the differentiation of Th17 helper cells and Treg cells in human T cells *in vitro* [11]. An earlier study showed that applying the ACC1 inhibitor soraphen A could restrain Th17 cell differentiation and their proliferative ability while promoting the development of Treg cells *in vitro* and *in vivo* in an experimental autoimmune encephalomyelitis model in mice [6]. ACC1 deletion also suppresses Th17 immune response, as well as infiltration of CD4^+^ T cells into the lamina propria in murine models of colitis [12]. Further evidence is found in subsequent studies that ACC1 inhibition could decrease the binding of RORγt and IL-17, followed by the disruption of RORγt and p300 co-localization, eventually resulting in an impaired Th17 differentiation [13]. As for CD8^+^ T cell populations, Lee *et al*. showed that specific deletion of ACC1 rendered CD8^+^ T cells incapable of blasting, proliferating, and surviving [14]. Akin to the ACC1 enzyme, FASN also catalyzes the synthesis of fatty acids and is critical for the autoimmune inflammatory function of Th17 cells [15]. FASN contributes to the maturation of Treg cells and is required for Treg cell-mediated tumor growth, though its role in Treg cell-mediated immune homeostasis is dispensable [16].

### Fatty acid uptake

Except for hepatocytes and adipocytes that rely on *de novo* lipogenesis, most tissues take up exogenous fatty acids for their energy needs, highlighting the necessity of cellular fatty acid uptake in physiological settings [17]. The uptake, transport, and trafficking of fatty acids across the plasma membrane are mediated by several well-identified membrane proteins, including CD36, solute carrier protein family 27 (SLC27), and fatty acid-binding proteins (FABPs) 4 and 5 [17–19]. For naïve CD4^+^ T cells, the extrinsic T-cell receptor-mammalian target of rapamycin complex 1 (TCR-mTORC1) axis activates the peroxisome proliferator-activated receptor gamma (PPARγ) and further elevates the expression of fatty acid uptake-associated genes, like FABP5. This process is essential for the early activation and rapid proliferation of CD4^+^ T cells upon TCR stimulation [20]. The participation of FABP5 has also been investigated in Treg cells. FABP5 functions to prevent the release of mitochondrial DNA into the cytosol and thus the activation of type I interferon (IFN) signaling and heightened IL-10 production, thus attenuating the immunosuppressive ability of Treg cells [21]. Therefore, this study indicates that FABP5 has an adverse effect on Treg cells. In contrast, FABPs play a positive role in CD8^+^ tissue-resident Tmem cells (Trm cells) considering that FABPs bolster their formation and survival [22]. It is also worth mentioning that recently several papers have uncovered the crucial roles of CD36 and FABP5 in intratumoral T cells, expediting our understanding of fatty acid uptake and cancer immunosurveillance. This part will be further illustrated in the section below that highlights the TME.

### Fatty acid oxidation

In contrast to fatty acid synthesis and uptake, fatty acid oxidation (FAO, also called β-oxidation) is a catabolic process that breaks down fatty acids to acetyl-CoA to produce energy or provide materials for diverse biochemical reactions. At the beginning of this process, free fatty acids are converted into acyl-CoA by acyl-CoA synthetase. Next, the long fatty acid-acyl-CoA is shuttled into the inner mitochondrial membrane by carnitine palmitoyltransferase 1 (CPT1), which is a rate-limiting step. After being transferred into the mitochondrial matrix, long-chain acyl-CoA undergoes β-oxidation consisting of four steps: (i) dehydrogenation catalyzed by acyl-CoA dehydrogenase; (ii) hydration catalyzed by enoyl-CoA hydratase; (iii) dehydrogenation catalyzed by 3-hydroxy acyl-CoA dehydrogenase; and (iv) thiolytic cleavage catalyzed by beta-keto thiolase [23]. The reaction is repeated until the fatty acid is fully converted into acetyl-CoA, which is further utilized by the tricarboxylic acid cycle and oxidative phosphorylation (OXPHOS) or by other biochemical reactions.

As of now, specific FAO requirements vary based on the type of T cell. In general, naïve T cells utilize FAO and OXPHOS for the maintenance of their quiescent state and rely on glycolysis for their activation, differentiation, and growth [24]. Teff cells tend to decrease FAO upon activation, while generation of Treg cells requires extensive levels of FAO. For instance, a seminal study has delineated the metabolic programs in various T cell subsets, with Th1, Th2, and Th17 subsets being primary glycolytic and lipid synthetic, whereas Treg cells are mainly catabolic [25]. Additionally, Treg cells express elevated levels of CPT1a and cytochrome *c* compared to Th17 cells, indicating greater FAO and electron transport chain activity in Treg cells during their differentiation [26]. To further understand why Treg cells have a preference for FAO, several studies investigated the role of Forkhead box P3 (Foxp3), a master regulator of Treg cells, and found that Foxp3-driven FAO could fuel OXPHOS and protect Treg cells from long-chain fatty acid-mediated cell death [27]. Moreover, Foxp3 suppresses the transcription of Myc and glycolysis in low-glucose, high-lactate milieus [28]. Consequently, these results suggest that Foxp3 might reprogram the metabolic control in Treg cells to confer a resistance to a hostile tissue environment.

Apart from modulating Treg cells, FAO has also been widely acknowledged to promote and sustain the immune function of CD8^+^ Tmem cells, which are featured by their long-lived persistence, as well as a quiescent, non-proliferative state [29]. In terms of the sources of fatty acids for FAO, Tmem cells make use of extracellular glucose rather than extracellular fatty acids to support FAO. Further, no lipid droplets are detected in Tmem cells, suggesting that they synthesize enough substrates required for FAO and rely on the intrinsic lysosomal acid lipase to mobilize fatty acids for FAO [5]. In line with this finding, IL-7 facilitates glycerol import into Tmem cells for subsequent fatty acid esterification, triglyceride synthesis, and FAO [30].

Several mechanistic studies have been conducted to understand how Tmem cells undergo FAO to sustain their energy. For instance, tumor necrosis factor (TNF) receptor-associated factor 6 promotes Tmem cell generation via stimulating AMP-activated protein kinase (AMPK) and blocking mTOR cascades, thereby increasing FAO [31]. In addition, Tmem cells display a substantial mitochondrial spare respiratory capacity, and this process is modulated by IL-15. IL-15 drives mitochondrial biosynthesis in CD8^+^ T cells, accompanied by the increased expression of CPT1a and elevation of FAO, which ensure a higher amount of mitochondrial mass, thus enabling CD8^+^ Tmem cell longevity [32]. Accordingly, upon programmed death-1 (PD-1) ligation, ­activated T cells acquire the metabolic and bioenergetic properties resembling those of Tmem cells, including increased expression of CPT1a and enhanced FAO [33]. Nevertheless, existing evidence using specific genetic deletion of the gene encoding CPT1a in T cells shows that the ACC2−CPT1a axis is dispensable for the differentiation and formation of Teff, Treg, and Tmem cells [34]. This study also refutes previous findings by demonstrating the potential off-target effects of the CPT1a inhibitor etomoxir, and as in this study, 3 μmol/L etomoxir rather than higher doses of etomoxir (>100 μmol/L) was applied [34]. Of note, etomoxir causes a dose-dependent inhibition of proliferation by Treg cells and Th17 cells independent of CPT1a expression [34]. However, this study could not rule out the participation of FAO in Tmem and Treg cells, and it could only hint at the presence of other pathways or mechanisms that may be involved in CPT1a-induced FAO and OXPHOS enhancement. Therefore, a more elaborate role for CPT1a in T cell functions should be further evaluated.

## Cholesterol homeostasis

Cholesterol is an essential lipid for mammalian cells. It serves as an essential component of the plasma membrane, as well as a precursor for steroid hormones, bile acids, and vitamin D. Metabolites of cholesterol also function as signal transducers for innate and adaptive immune responses [35]. Similar to fatty acid metabolism, intracellular cholesterol homeostasis is tightly controlled by cholesterol synthesis, import, excretion, and esterification [36]. Moreover, these processes are closely governed by two classes of master transcriptional regulators: namely, sterol regulatory element–binding proteins (SREBPs) and liver X receptors (LXRs), with the former facilitating cholesterol accumulation and biosynthesis and the latter facilitating cholesterol efflux [37, 38]. High levels of cholesterol in the blood can disturb T cell homeostasis and are prone to cause a T cell-mediated inflammatory response [39]. Therefore, elaborate and stringent regulation of cholesterol levels are required to determine T cell fate in different physiological and pathological settings.

### Cholesterol *de novo* synthesis

Biosynthesis of cholesterol is mainly controlled by the mevalonate pathway. The initial step is to condense two acetyl-CoA molecules into acetoacetyl-CoA for further synthesis of 3-hydroxy-3-methylglutaryl-CoA (HMG-CoA). Conversion of HMG-CoA to mevalonic acid requires HMG-CoA reductase (HMGCR), which is also the key rate-limiting enzyme during the entire process of cholesterol synthesis and is the key molecular target of statin medications used to lower serum cholesterol levels. Next, mevalonic acid undergoes a conversion into several important isoprenoid intermediates, including farnesyl pyrophosphate and geranylgeranyl pyrophosphate (GGPP), followed by the Bloch pathway, the Kandutsch-Russell pathway or a hybrid pathway before its final conversion to cholesterol [40]. HMGCR is activated by sterol regulatory element-binding protein 2 (SREBP2) upon low cholesterol concentrations, and both HMGCR and SREBP2 are devoted to different T cell functions. Therefore, the biological functions of these lipogenesis targets have been widely investigated in T cells. A pivotal study has shown that upon antigen stimulation, SREBP activity contributes to CD8^+^ T cell blastogenesis, proliferation, and survival by modulating a set of genes related to lipid anabolism. Furthermore, addition of exogenous cholesterol restores the proliferation of Teff cells upon SREBP deficiency, indicating that adequate cholesterol biosynthesis induced by SREBP benefits T cell activation and proliferation [41]. During various T cell differentiation stages, different T cell clusters display distinct metabolic requirements in response to immunological signals. For example, mTOR1, the downstream target of the PI3K and AKT pathways and the upstream regulator of SREBP2, is responsible for determining the cell fate of CD4^+^ and CD8^+^ T cells [42]. T cells with high amounts of mTOR are destined to become Teff cells (e.g. Th1 and Th17 cells), while the others become long-lived Tmem cells or Treg cells [42–44]. However, it is worth noting that though naïve cells need lower mTOR levels for differentiation into Treg cells, the suppressive functions of Treg cells are sustained by high mTOR/Raptor-cholesterol levels. Mechanistically, mTOR1 coordinates Treg cell functional competency by promoting the mevalonate pathway, upregulating the expression of cholesterol biosynthesis genes, and increasing the levels of the suppressive molecules cytotoxic T lymphocyte antigen 4 (CTLA4) and inducible T-cell costimulator (ICOS) [45]. Similarly, the activated mevalonate pathway regulated by liver kinase B1 (LKB1) demonstrates positive regulation of mevalonate genes on Treg cell activity and survival, highlighting the importance of cholesterol metabolism in immune homeostasis and tolerance [46].

### Cholesterol uptake

Besides *de novo* synthesis, cells also take up cholesterol from the diet or blood to main their cholesterol homeostasis. The process is mainly mediated by Niemann–Pick type C1 (NPC1)-like 1 (NPC1L1), which absorbs cholesterol from the intestinal lumen or by the low-density lipoprotein (LDL) receptor (LDLR), which takes up cholesterol from the blood [36]. LDLR has a high binding affinity for apolipoprotein E (ApoE) to promote cholesterol packing and transportation into the blood [47]. The deficiency of ApoE causes severe hypercholesterolemia and boosts CD4^+^ T cell activation. Mechanistic studies have further shown that ApoE knockout mice significantly accumulate the cholesterol and oxysterols in dendritic cells (DCs), thus increasing DC antigen presentation and enhancing inflammatory cytokine secretion in T cells [48, 49]. There also exists a negative regulator of LDLR, named proprotein convertase subtilisin/kexin type 9 (PCSK9), which promotes the degradation of LDLR and consequently blocks cholesterol uptake [50]. The importance of the PCSK9-LDLR axis in T cells will be delineated below in the section highlighting the TME.

### Cholesterol esterification and efflux

The homeostasis of cholesterol within cells is maintained dynamically as excess cholesterol that accumulates by biosynthesis or uptake is actively removed by efflux from cells or is stored intracellularly as cholesteryl esters in lipid droplets [51]. Hence, cholesterol efflux mediated by the activation of LXR, as well as its target genes like ATP-binding cassette subfamily A member 1 and G member 1 (ABCA1/G1), is necessary for the balance of cholesterol biomass, and the functions of the LXR-ABCA1-ABCG1 pathway in T cells have been investigated. A pioneering study showed that upon T cell activation, the SREBP pathway is induced while the LXR pathway is repressed [41, 52]. Mechanistically, LXR inhibition upon TCR stimulation is partially ascribed to the oxysterol metabolizing enzyme Sulfotransferase 2B1 (SULT2B1), which catalyzes the transfer of sulfate groups to oxysterols to inhibit their ability to act as LXR ligands. This inhibition is necessary as otherwise ligand-activated LXR modulates ABCG1 expression, resulting in greater efflux of cholesterol such that proliferating T cells cannot acquire sufficient sterols for their energy needs, thus limiting their expansion [52]. Via a different mechanism, another study supported the immune-suppressive role of LXR in primary human CD4^+^ T cells by showing it remodels the plasma membrane lipid composition (increasing glycosphingolipid and decreasing cholesterol levels), lowering the plasma membrane stability and altering immune synapse formation [53]. In addition, ligand-mediated activation of LXR suppresses the promotion of MAP kinase and nuclear factor-kappaB (NF-κB) signaling in an ABCA1-dependent manner [54]. However, apart from an immune-suppressive function, LXR can also execute pro-inflammatory roles. For example, a recent study showed that other than directly influencing T cells, LXR agonism depletes myeloid-derived suppressor cells (MDSCs) by upregulating ApoE. Importantly, this effect is associated with enhanced cytotoxic T lymphocyte responses in both mice and patients [55]. In addition to cholesterol efflux, conversion of free cholesterol to a less toxic cholesteryl ester by acyl-coA:cholesterol acyltransferases (ACAT) is another method to store or secrete cholesterol [56]. Inhibition of ACAT1 profoundly increases the plasma membrane cholesterol levels and assists the CD8^+^ T cell killing effects [57], which will be described in detail later.

Collectively, fatty acid metabolism and cholesterol metabolism tune the immune response of T cells by modulating the energy and nutritional demand between distinct T cell clusters. To further clarify, naïve T cells rely on OXPHOS for basic bioenergetic needs. Once T cells switch from quiescence to activation, they need higher and faster energy and nutrient supplies. These high energy and nutrient demands are mainly fulfilled by elevated levels of glycolysis, as well as by strengthened synthesis and uptake of fatty acids and cholesterol [58]. Following the rapid growth phase, T cells proliferate, and subsequently differentiate into various functional groups, among which Th cells and cytotoxic T cells sustain heightened lipid biomass whereas Treg and Tmem cells require enhanced FAO. Paradoxically, though the differentiation of naïve T cells towards Treg cells requires lower lipid synthesis and uptake, the immune-suppressive function of Treg cells is sustained by high lipid content.

## The role of lipid metabolism in tumor-infiltrating T cells

**Figure 1:**
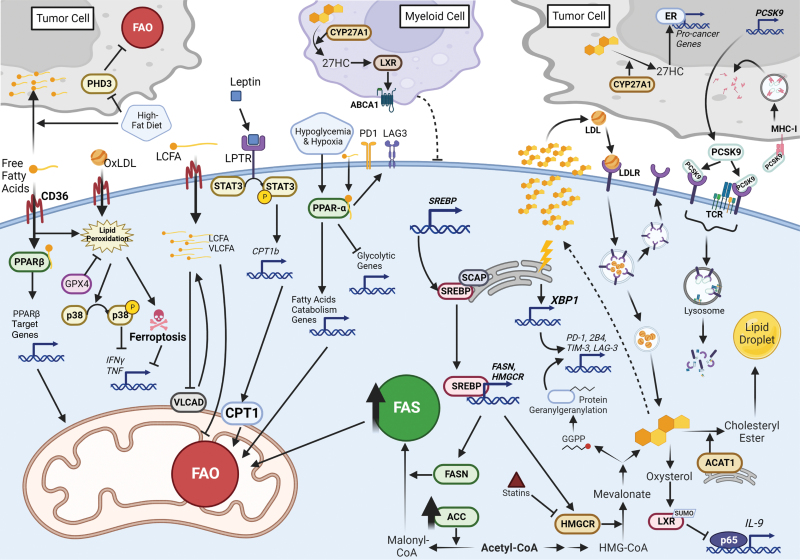
Lipid metabolism in tumor-infiltrating T cells. Accumulated fatty acids are commonly found in the TME of different cancer types, in the form of free fatty acids or LDL particles. T cells take up these fatty acids via the membrane protein CD36. Upregulated CD36 expression in intratumoral Treg cells enables these cells to take up more fatty acids, which further activates the PPARβ pathway, supporting mitochondria fitness in Treg cells. In contrast, in tumor-infiltrating Teff cells, the TME-cholesterol-induced CD36 upregulation leads to increased intracellular fatty acids levels and enhanced lipid peroxidation and ferroptosis, eventually resulting in reduced cytotoxic cytokine production and antitumor ability of intratumoral Teff cells. Uptake of OxLDL in the TME can induce lipid peroxidation and the downstream activation of p38, leading to dysfunction of CD8^+^ TILs, as well. FAO is also a key process involved in T cell metabolism though its role in the tumor-infiltrating Teff cells is still controversial. Upon hypoxia and hypoglycemia dual-stress, CD8^+^ TIL preserves its energy production and effector functions by enhancing PPARα signaling and fatty acid catabolism. However, in a murine pancreatic cancer model, LCFAs impair mitochondria function and repress FAO in CD8^+^ TILs, while a downregulation of very-long-chain acyl-CoA dehydrogenase (VLCAD) further aggravates the accumulation of LCFAs and very-long-chain fatty acids (VLCFAs) in cells. On the contrary, elevated fatty acids in obesity induce leptin secretion in the TME, which triggers activation of STAT3 and expression of CPT1b, thereby promoting FAO while repressing glycolysis and the antitumor function of CD8^+^ TIL. As in the HFD TME, tumor cells but not infiltrating CD8^+^ T cells adapt to such a fatty acid-enriched environment via repression of the expression of PHD3, which controls FAO. Although the mechanism is still unknown, such a reduction of PHD3 enables tumor cells to create a cold TME, leading to the dysfunction of infiltrated CD8^+^ T cells. Regarding the cholesterol metabolism in the TME, expression of SREBP in intratumoral Treg cells allows them to augment the expression of FASN and key enzyme for cholesterol synthesis, including HMGCR. Upregulated ACC2 and FASN promote fatty acid synthesis which provides more fuel for FAO, supporting Treg cell survival in the TME. In CD8^+^ TILs, upregulated HMGCR leads to both increased GGPP and cholesterol. GGPP induces protein geranylgeranylation whereas accumulation of cholesterol induces ER stress and XBP1 expression, eventually enhancing CD8^+^ TILs exhaustion. In addition, CD8^+^ T cells that produce IL-9, named Tc9 cells, exert better antitumoral function. Addition of cholesterol into cell culture diminish the effect of Tc9 cells, as cholesterol can be further converted to oxysterol which activates LXR via sumoylation, leading to reduced p65 binding to the IL-9 promoter. Cholesterol can also be esterified by ACAT1, while inhibition of ACAT1 elevates CD8^+^ T cell effector function. CYP27A1 mediates the synthesis of another cholesterol derivative, 27-HC. In tumor cells, 27-HC activates ER, leading to expression of pro-cancer genes; in myeloid cells, 27-HC promotes LXR function and expression of ABCA1, contributing to the anti-immune effect of myeloid cells. Cells take up cholesterol-containing LDL via LDLR-mediated transport vesicles. In the TME, tumor cell-secreted PCSK9 binds to LDLR, blocking LDLR-TCR clustering and signaling. Autocrine PCSK9 also promotes the degradation of MHC-I in tumor cells, thus causing the defects of antitumor functions of CD8^+^ TILs.

The TME is a niche that consists of distinct cell types, including cancer cells, immune cells, and stromal cells [59]. Cancer cells hijack nutrients, such as glucose, amino acids, and lipids, to sustain their energetic needs, thereby promoting tumor progression [60]. Meanwhile, the cell fate and function of T cells also rely on the nutrient supply. Therefore, the dynamic interaction between the tumor and host within the TME makes the metabolic demand for cancer cells and immune cells more intricate. Among those metabolites, lipids are one of the prominent components but have often received less attention. Moreover, hypoglycemia and hypoxia in the TME reshape the lipid metabolism requirements of different immune cell types. Thus far, numerous studies have been devoted to investigating the correlation between lipid compositional changes and dysfunction of tumor-infiltrating lymphocytes (TILs) in the TME (Table 1 and Fig. 1).

**Table 1:** Lipid metabolism in tumor-infiltrating T cells

	Findings	Function in intratumoral T cells	Target involved	Indication	References
**Fatty acid synthesis**	Treg cells confer proliferative survival advantage by increasing fatty acid synthesis rather than fatty acid uptake in the TME.	Intratumoral Treg Cells (+)	ACC2	Colon cancer/Melanoma	[61]
**Fatty acid uptake**	Intratumoral Treg cells upregulate CD36 expression to support mitochondrial fitness via a PPARβ-dependent mechanism. Genetic ablation of *CD36* in Treg cells selectively abrogates the abundance and suppressive activity of intratumoral Treg cells.	Intratumoral Treg Cells (+)	CD36	Colon cancer/Melanoma	[63]
	The TME is enriched with lipids and oxidized lipids. CD36-mediated uptake of lipids in CD8^+^ T cells leads to increased lipid peroxidation and induces CD8^+^ T cell dysfunction.	Intratumoral CD8^+^ T cells (–)		Colon cancer/Melanoma	[64]
	TME-cholesterol-induced CD36 expression in tumor-infiltrating CD8^+^ T cells promotes uptake of fatty acids in tumor-infiltrating CD8^+^ T cells and induces lipid peroxidation and ferroptosis, eventually leading to impaired antitumor ability.	Intratumoral CD8^+^ T cells (–)		Melanoma	[65]
	Defect of FABP5 limits the commitment of pDCs and Treg cells in the TME.	Intratumoral Treg Cells (+)	FABP5	Melanoma	[67]
**FAO**	Accumulation of LCFAs caused by downregulated VLCAD impairs the mitochondrial function of CD8^+^ T cells and reduces lipid catabolism in pancreatic ductal adenocarcinoma. Overexpression of VLCAD improves the survival and persistence of CD8^+^ T cells in the TME.	Intratumoral CD8^+^ T cells (+)	VLCAD	Pancreatic ductal adenocarcinoma	[68]
	CD8^+^ TILs experiencing both hypoxia and hypoglycemia within the TME enhance PPARα signaling and fatty acid catabolism to preserve their energy production and effector functions.	Intratumoral CD8^+^ T cells (+)	PPARα	Melanoma	[69]
	Elevated fatty acids in obesity inhibits CD8^+^ Teff cell glycolysis through the leptin-PD-1-STAT3-FAO pathway, thereby restricting CD8^+^ T cell antitumor functions.	Intratumoral CD8^+^ Teff cells (–)	STAT3	Breast Cancer	[70]
	A high-fat diet (HFD) accelerates colon tumor growth via impairing CD8^+^ T cell by repressing tumor cell Prolyl Hydroxylase-3 (PHD3) expression, which controls FAO availability in the HFD TME.	Intratumoral CD8^+^ Teff cells (unclear)	PHD3	Colon cancer	[71]
	Tissue-resident Tmem cells utilize mitochondria FAO to meet their energy requirements and prolong their survival.	Intratumoral CD8^+^ Trm cells (+)	/	Gastric cancer	[66]
***De novo* cholesterol synthesis**	In the TME, HMGCR-dependent cholesterol synthesis induces CD8^+^ TIL exhaustion in an ER-stress-XBP1-dependent manner.	Intratumoral CD8^+^ T cells (–)	HMGCR	Colon cancer/Myeloma/Melanoma	[80]
	Inhibiting SREBP-dependent lipid synthesis by deleting SCAP in Treg cells unleashes an effective antitumor immune response. SCAP/SREBP signaling and PD-1 function in intratumoral Treg cells are important for the proper control of PI3K signaling and IFNγ production.	Intratumoral Treg cells (+)	SCAP/SREBP	Colon cancer/Melanoma	[16]
**Cholesterol uptake**	Tumor-derived PCSK9 inhibits LDLR and the TCR signaling of tumor-infiltrating CD8^+^ T cells and inhibits the antitumor activity.	Intratumoral CD8^+^ T cells (+)	PCSK9-LDLR	Colorectal cancer/Lung cancer/Breast cancer/Melanoma	[85]
	Autocrine PCSK9 promotes the degradation of MHC-I in tumor cells to reduce tumor-infiltrating T cell efficiency.	Intratumoral CD8^+^ T cells (+)		Colon cancer/Breast cancer/Melanoma	[86]
**Cholesterol esterification and efflux**	Reducing cholesterol enhances CD8^+^ Tc9 cell persistence and potentiates its antitumor activity *in vivo*. Cholesterol or its derivatives inhibit IL-9 expression by activating LXRs, leading to LXR-sumoylation and reduced p65 binding to IL-9 promoter.	/	LXR	Colorectal cancer/Melanoma	[81, 82]
	Genetic ablation or pharmacological inhibition of ACAT1 increases T cell receptor clustering and immune synapse formation and elevates CD8^+^ T cell effector function.	Intratumoral CD8^+^ T cells (–)	ACAT1	Melanoma	[57]
	27-HC, which is synthesized with CYP27A1 and released in an autocrine manner by myeloid cells, works on myeloid cells through LXR-ABCA1 to suppress cytotoxic CD8^+^ T cell activity, eventually promoting breast cancer progression.	Intratumoral CD8^+^ T cells (–)	CYP27A1	Breast Cancer	[88, 89]

### Role of fatty acid metabolism in T cells in the TME

The TME is a lipid-rich locus that benefits tumor cell growth and impairs T cell function. Therefore, to understand the fatty acid determinants of TME-mediate immune suppression, a series of studies were conducted. Under tumor-associated stimuli, Treg cells enlarge their lipid pool to guarantee their expansion and sustain their suppressive function. Nevertheless, the proliferation and lipid accumulation ability of Treg cells is abolished when they are treated with the ACC inhibitor 5-tetradecyloxy-2-furoic acid (TOFA), which disrupts the fatty acid synthesis cascade, indicating the reliance of intratumoral Treg cells on fatty acid synthesis. Importantly, glycolysis is preferentially preserved in tumor-infiltrating Treg cells, and it fuels the fatty acid ­biosynthesis pathway. Hence, the circuitry among glycolysis, fatty acid synthesis, and FAO comprises an immunosuppressive pathway [61]. In addition to *de novo* synthesis, uptake of fatty acids by CD36, a scavenger receptor involved in fatty acid translocation, has been demonstrated to modulate diverse sets of T cells [62]. Intratumoral Treg cells upregulate CD36 to preserve their activity while intratumoral cytotoxic CD8^+^ T cells are depleted by CD36-mediated ­oxidized lipid uptake that leads to lipid peroxidation and ferroptosis [63–65]. In this regard, CD36 may serve as a promising anti-cancer target. By contrast, in gastric adenocarcinoma, elevated CD36 expression and increased lipid content are important to the survival of CD8^+^ Trm cells, indicating the positive role of CD36 in antitumor responses [66]. The differential effects of CD36 on T cells may be attributed to the traits of Tmem cells, as effector CD8^+^ T cells are prone to deposit fatty acids, while Trm cells are prone to turn glucose into fatty acids and immediately break down fatty acids via FAO. In addition, the deficiency of the fatty acid cytosolic transporter FABP5 hinders the generation of intratumoral Treg cells by regulating plasmacytoid DCs (pDCs), suggesting the complex crosstalk between different immune cells within the TME [67]. To date, our understanding of the dependency of CD8^+^ TILs on FAO and OXPHOS is still not clear. In pancreatic ductal adenocarcinoma, LCFAs progressively accumulate and dysregulate mitochondrial function, as well as fatty acid catabolism of CD8^+^ T cells, thus inducing severe lipotoxicity in intrapancreatic CD8^+^ T cells that contributes to their exhaustion [68]. In addition, under the harsh condition of glucose and oxygen deficiency, CD8^+^ TILs isolated from melanoma samples combat tumor cells by upregulating PPARα signaling and enhancing FAO to fuel their energetic demands [69]. These findings indicate that promoting fatty acid catabolism facilitates the effector functions of T cells in the TME. However, strengthened FAO may also dampen the viability of CD8^+^ Teff cells. In the context of the obesity-related breast TME, the leptin-Signal transducer and activator of transcription 3 (STAT3) axis promotes FAO and inhibits glycolysis of CD8^+^ Teff cells. Both *Stat3* functional knockout and FAO inhibition enhance the accumulation, proliferation, and function of tumor-infiltrating Teff cells, suggesting that STAT3 diminishes CD8^+^ Teff cell functions via promoting FAO [70]. It has been also reported that in high-fat–induced MC38 colon tumors, tumor cells and T cells in the TME reprogram their metabolism to take up more fatty acid for oxidation; however, the panel of FAO-related genes is not altered in these colon tumor CD8^+^ TILs. As the mammary fat pads are full of adipocytes, these findings suggest that obesity-regulated lipid metabolism in tumor-infiltrating T cells might be cancer-type-dependent [71]. Unlike tumor-infiltrated Teff cells, which show a controversial dependency on strengthened FAO, CD8^+^ Trm cells in the TME of gastric adenocarcinoma are prone to utilize FAO for their survival and longevity, mediating better antitumoral immunity. This finding is consistent with a previous result showing that skin CD8^+^ Trm cells increase FABP4 and FABP5 expression to promote the uptake of exogenous fatty acids and their oxidation to orchestrate Trm cell persistence [66, 72]. It is worth mentioning that in different stages of exhausted T cells, the lipid metabolism pattern also differs. From progenitor to terminal exhaustion, T cells show higher expression of PD-1 [73, 74]. As a result of PD-1 ligation, CPT1a and adiposite triglyceride lipase (ATGL) are upregulated, leading to the promotion of FAO in terminally exhausted T cells [33, 75]. In addition, chronic IFN signaling has been shown to promote CD8^+^ T cell exhaustion via increasing fatty acid uptake and FAO [76]. Meanwhile, mitochondria also undergo reduced membrane potential and increased levels of lipid peroxidation during T cell exhaustion [77–79]. Taken together, fatty acid metabolism fine tunes T cell responses and mediates their antitumor activity in diverse ways within different contexts.

### The role of cholesterol metabolism in T cells in the TME

Apart from fatty acid accumulation, cholesterols and cholesterol esters are also enriched in the TME, even though the effect of cumulative cholesterols on T cells remains to be determined. It has been proposed that high cholesterol content in the TME causes CD8^+^ TIL exhaustion by upregulating the expression of several immune checkpoint proteins, including PD-1, 2B4, T-cell immunoglobulin, mucin domain-3 (Tim-3), and lymphocyte Activation Gene-3 (LAG-3) [80]. The study further elucidated the mechanism behind this phenotype and found that cholesterol could trigger the endoplasmic reticulum (ER) stress sensor X-box binding protein 1 (XBP1) to increase the levels of these immune checkpoint proteins and decrease the expression of IFN-γ and Granzyme b (Gzmb), as well as reducing the proliferative capability of CD8^+^ T cells [80]. Moreover, adoptive cell therapy shows that CD8^+^ T cells that produce IL-9 (Tc9 cells) tend to have better persistence and antitumor responses against a B16 melanoma model than type 1 cytotoxic CD8^+^ T cells (Tc1 cells) [81]. However, this superior effect of Tc9 cells could be diminished by adding cholesterol to the cell culture, as cholesterol derived-oxysterols inhibit IL-9 secretion by enhancing LXR-sumoylation [82]. As these studies noted, high cholesterol adversely affects the cytotoxicity of T cells. Contrary to this notion, one previous study pointed out that increased cholesterol levels in the plasma membrane could promote the immunological synapse formation and TCR clustering of CD8^+^ T cells, which orchestrates their production of cytolytic granules and inflammatory cytokines [57]. Furthermore, a natural analog of cholesterol, cholesterol sulfate, can restrain CD3 immunoreceptor tyrosine-based activation motif phosphorylation, further supporting the notion that cholesterol spatially coordinates the membrane microenvironment and T cell signaling [83]. Together, the discrepancy of insights on cholesterol function in the TME may be attributed that to the location of cholesterol as intracellular and plasma membrane cholesterol might exert different functions. In addition, reinforcement of intrinsic cholesterol in T cells may augment their antitumor response, while overload of exogenous cholesterol causes T cell exhaustion.

In a further effort to understand how the TME reprograms the cholesterol metabolic fitness of CD8^+^ TIL cells, cholesterol synthesis and uptake pathway-related genes were analyzed. As previously mentioned, LDLR is a protein involved in cholesterol-containing lipoprotein trafficking, and PCSK9 is a vital post-translational regulator of LDLR levels [84]. A recent study observed the comparative low level of LDLR in total CD8^+^ TILs compared to active CD8^+^ TILs, indicating the potential necessity of LDLR for CD8^+^ T cells effector function [85]. Further analysis revealed that apart from canonical LDL and cholesterol uptake, LDLR also modulates TCR recycling and signaling, though this signaling transduction enhancement of T cell function could be inhibited by tumor-derived PCSK9. Therefore, blockade of the PCSK9-LDLR-TCR axis confers antitumor activity on intratumoral CD8^+^ T cells [85]. Similar research has been conducted in cancer cells and found that PCSK9 inhibition could restore the levels of surface MHC-I of cancer cells to guarantee antigen presentation and CD8^+^ lymphocytic infiltration [86]. Despite the distinct mechanisms and working models, these two studies have uncovered the cholesterol metabolic program in driving tumor-infiltrating T cells in the TME, and they also have drawn the same conclusion that the cholesterol uptake-associated protein PCSK9 serves as an auspicious therapeutic target. In fact, anti-PCSK9 therapies (e.g. Repatha) have already been developed and used in the clinic to treat cardiovascular disease and thus could be repurposed to target cancer [87].

Apart from the crosstalk between cholesterol and Teff cells in the tumor milieu, the adaptation of cholesterol metabolism by immunosuppressive T cells subsets like Treg cells has also been evaluated. It has been reported that the viability and TCR-induced functional maturation of Treg cells rely on the upregulation of SREBP signaling within the TME, as deficiency of SREBP-cleavage-activating protein (SCAP) (a protein involved in cholesterol biosynthesis) or FASN in Treg cells retards the tumor growth of MC38 colon adenocarcinoma and B16 melanoma tumors [16]. Moreover, PD-1 expression is reduced in SCAP-deficient Treg cells, and a mechanistic study showed that the mevalonate pathway induces protein geranylgeranylation, which further promotes PD-1 expression in Treg ells, establishing a linkage between cholesterol synthesis and immune checkpoint signaling in antitumor immunity [16].

In addition, cholesterol metabolites, such as 27-hydroxycholesterol (27-HC), have been shown to promote breast cancer metastasis by recruiting and activating polymorphonuclear neutrophils and γδ T cells and decreasing cytotoxic CD8^+^ T cells within tumors [88]. The conversion of cholesterol to 27-HC requires the rate-limiting enzyme Cholesterol 27-hydroxylase (CYP27A1). Thus, both genetic depletion and pharmacological inhibition of CYP27A1 reduce breast cancer metastasis under high-fat-high-cholesterol diet conditions [89]. Echoing this study, the same group further showed the connection among 27-HC, T cells, and macrophages in the TME, as they showed that 27-HC-treated macrophages impair T cell expansion in an LXR-dependent manner and shape an immunosuppressive environment [89].

In summary, the balance between intracellular and extracellular cholesterol content might be vital for determining the cell fate and tumoricidal effects of T cells in the TME. Importantly, reciprocal regulation among different immune populations also should be taken into consideration when the functions of TILs are being accessed.

## Targeting lipid metabolism in T cells for optimal cancer immunotherapy

Overall, the tumor immunosuppressive niche is characterized by the glucose- and oxygen-deprived environment, which easily leads to the dysfunction of tumor-infiltrating Teff cells. The exhaustion and low persistence phenotype of T cells in this environment is partially attributed to the lipid metabolic programming. Therefore, rewiring lipid metabolism in T cells may help to rejuvenate inflammatory T cells against cancer (Table 2).

**Table 2:** Anti-cancer interventions that target lipid metabolism in T cells

Strategy	Pathway	Target	Agent	Combination	T cells affected	Mechanism of action	Indication	References
**Targeting fatty acid metabolism**	Fatty acid uptake	CD36	anti-CD36 mAb	anti-PD-1 antibody	Intratumoral Treg cells	Inhibited excessive fatty acid uptake, prevented the ferroptosis of CD8^+^ T cells, and suppressed	Yumm1.7 melanoma	[65]
					Intratumoral Teff cells	Treg functions	B16 melanoma	[63]
	FAO	PPARα	Bezafibrate	anti-PD-L1 antibody	CD8^+^ T cells	Facilitated PPARα-FAO axis to enhance Teff cells survival and anti-tumor function	MC38 colon cancer	[92]
		PPARα	aCD3/F/AN	/	CD8^+^ T cells	Increased lipid uptake, increased mitochondrial membrane, enhanced CD8^+^ T cell infiltration, and IFN-γ and granzyme B production	B16F10 melanoma	[106]
		CPT1/STAT3/leptin	Perhexiline/CTLA4 (apt)-Stat3 siRNA/anti-leptin antibody	/	Intratumoral Teff cells	Inhibited leptin-STAT3-CPT1-FAO axis and promoted Teff function under obesity	Obesity-promoted MMTV-PyMT breast cancer	[70]
**Targeting cholesterol metabolism**	Cholesterol *de novo* synthesis	HMGCR	Statins	anti-PD-1 antibody	Th1 and cytolytic T cells	Decreased protein prenylation, augmented antigen presentation, and induced Th1 and cytolytic T cell response	B16-Ova melanoma, TC-1 cervical cancer	[98]
		AMPK	AICAR	anti-PD-1 antibody/anti-CTLA-4 antibody/HMGCR inhibitor	CD4^+^ T cells, CD8^+^ T cells, Treg cells	Downregulated PD-1 in Treg cells through the HMGCR/p38 MAPK/GSK3β axis	B16F10 melanoma, TC-1 cervical cancer, MC38 colon cancer	[96]
		PPARγ	Pioglitazone	alpha-galactosylceramide	invariant natural killer T cells	Increased IFN-γ production	B16F10 melanoma	[97]
	Cholesterol uptake	PCSK9	PF-06446846	anti-PD-1 antibody	CD8^+^ T cells	Inhibited tumor progression and extended mice survival	B16F10 melanoma, MC38 colon cancer	[85]
	Cholesterol efflux and esterification	LXR	SR9243	/	CD8^+^ T cells	Increased CD8^+^ T cell tumor infiltration, inhibited PD-1 expression, promoted DC migration, and suppressed MDSC and Treg populations	E0771, EMT6 breast cancer	[100]
			RGX-104GW3965	anti-PD-1 antibody	CD8^+^ T cells	Reduced MDSC abundance and increased cytotoxic T cell activation	B16F10 melanoma, Lewis lung carcinoma	[55]
		ACAT1	Avasimibe, CP-113,818, K-604	anti-PD-1 antibody/DOX–MNPs/PTX/αGC-TH-Lip	CD8^+^ T cells	Increased plasma membrane cholesterol levels, and enhanced T-cell receptor clustering and signaling	B16F10 melanoma, 4T1 breast cancer	[57, 101, 102]
		RORα	SR1078/cholesterol sulfate	/	CD8^+^ T cells	Increased IFN-γ production, and increased cancer cell death	HCT116 colon cancer	[103]
**Adoptive cell therapy**	Related to lipid metabolic adaption	PPARα/δ/β	GW501516	/	CD8^+^ T cells	Enhanced FAO and enhanced production of IFNγ and expression of T-bet	B16-gp33 melanoma	[105]
		PPARα	Fenofibrate	anti-PD-1 antibody	CD8^+^ T cells	Increased FAO, enhanced OXPHOS, and increased PD-1 and T-bet	B16(BRAF^V600E^) melanoma	[69]
		FOXP3	/	/	CD8^+^ T cells	Improved the cell proliferation, cytotoxicity, and tumor recruitment capability	B16 melanoma	[107]
		GPX4	ferroptosis inhibitor ferrostatin	/	CD8^+^ T cells	Decreased the lipid peroxidation in CD8^+^ TILs, and increased secretion of TNF and IFNγ	B16-gp33 melanoma	[64]
		RORα	LYC-54143	/	Th17 cells, T cytotoxic 17 cells (Tc17)	Potentiated the antitumor activity of human Th17 and Tc17 cells redirected with a CAR; made cells persist as long-lived memory cells *in vivo*	B16F10 melanoma, M108 mesothelioma	[109, 110]
		ACAT1	CAR-T-1847, CAR-T-1848	/	CD8^+^ T cells	Reduced tumor volume	MSLN-BxPC-3-Luci pancreatic carcinoma	[111]
			T-Tre/BCN-Lipo-Ava	/	CD8^+^ T cells	Enhanced cholesterol content and induced proportion of cytokine and cytolytic granule	B16F10 melanoma, LN-229 glioblastoma	[112]

### Targeting fatty acid metabolism to treat cancer

In the lipid-laden TME, appropriate lipid uptake and storage help to maintain Teff cell functions, whereas overloading of lipids might lead to their death. As noted above, CD36 impedes the function of tumor-infiltrating CD8^+^ T cells via the uptake of excessive fatty acids from the TME and the induction of lipid peroxidation and ferroptosis [64, 65]. By contrast, the intratumoral Treg cells benefit from CD36-PPARβ signaling and demonstrate dependency on CD36-mediated mitochondrial fitness and nicotinamide adenine dinucleotide (NAD^+^) levels. Therefore, anti-CD36 monoclonal antibody treatment reveals profound effects on inhibiting melanoma tumor growth in the recipient mice, accompanied by induction of Treg cell apoptosis, enhanced CD8^+^ T cell infiltration, and higher cytokine production in CD4^+^ and CD8^+^ TILs. Importantly, an anti-PD-1 mAb combined with anti-CD36 mAb or CD36^−/−^ Treg or CD36^−/−^ CD8^+^ T cells display additive effects in suppressing tumor growth [63, 65]. Other than decreasing the uptake of lipids, increasing the FAO pathway would also confer a survival advantage and the persistence of T cells, as exemplified by Tmem cells, which prevail over Teff cells under a harsh TME [90]. PPARα is a notable target considering its pivotal role in the transcriptional activation of FAO-related genes [91]. The tumoricidal effects of several PPARα agonists have already been extensively evaluated in different cancer types. For example, bezafibrate, a pan-PPAR agonist, can induce mitochondrial activation and FAO in CD8^+^ T cells *in vivo*, further promoting CD8^+^ T cell proliferation and effector function [92]. Upon PD-1 ligation, the glucose and amino acid utilization of activated CD4^+^ T cells is abrogated while the lipid catabolism rate of activated T cells is increased, suggesting a metabolism switching of T cells after PD-1 signaling [33]. These results hint that PD-1-induced FAO upregulation can inhibit the differentiation of activated T cells into Teff cells, thus promoting immune tolerance. Therefore, blockade of PD-1 might help to reinvigorate the effect of T cells. This hypothesis is also applicable to CD8^+^ T cells, as synergistic antitumor effects of combined PD-1 blockade and PPARα agonism (i.e. by fenofibrate or bezafibrate) are observed in several cancer models, including colon carcinoma, melanoma, and lung carcinoma [69, 92–94].

Even so, enhancing FAO is not always the optimal strategy, especially in high-lipid accumulation regions, like mammary adipocytes or fat tissues. In this case, STAT3, the transcription activator of CPT1a, attenuates the tumoricidal function of Teff cells and promotes tumor progression. By covalently linking a STAT3 siRNA and a CTLA4-specific aptamer, this conjugate successfully inhibits STAT3 activity, promotes tumor-infiltrating CD8^+^ T cell functions, and retards tumor growth of PyMT breast cancer under HFD feeding [70]. Moreover, both the CPT1 inhibitor perhexiline and an anti-leptin antibody are able to mitigate tumor growth by upregulating cytokine release by CD8^+^ T cells, indicating that targeting the leptin-PD-1-STAT3-FAO pathway represents a new direction in treating breast cancer [70, 95].

### Targeting cholesterol metabolism to treat cancer

Given the importance and necessity of cholesterol for cancer progression, therapeutics that block cholesterol pathways in cancer cells have been extensively reviewed [40]. Yet the strategies in treating cholesterol metabolism in T cells are to be further summarized here. To date, mevalonate-pathway inhibitors are feasible drugs that have been evaluated. Those inhibitors (i.e. 25-hydroxycholesterol, simvastatin, atorvastatin, and lovastatin) directly inhibit cholesterol biosynthesis and abrogate Treg cell proliferation and its effector molecule upregulation [45]. Other than direct functioning on cholesterol synthesis enzymes, molecules modulating upstream signaling pathways also exhibit considerable therapeutic potential. AMPK has been shown to inhibit both HMGCR and PD-1 expression in Treg cells. Therefore, 5-Aminoimidazole-4-carboxamide ribonucleoside (AICAR), a commonly used pharmacological activator of AMPK, exhibits synergistic antitumor effects when combined with statins or anti-PD-1 treatment [96]. Moreover, it has been reported that PPARγ promotes the expression of SREBP1, thereby enhancing cholesterol synthesis and IFN-γ production in iNKT cells. The combination of the PPARγ agonist pioglitazone and alpha-galactosylceramide bolsters IFN-γ production and invariant natural killer T (iNKT) cell-mediated antitumor immune responses [97]. In terms of targeting cholesterol uptake, PCSK9 is an ideal target as it hampers CD8^+^ T cell antitumor activity via LDLR and TCR signaling inhibition. Treatment of xenografts using PCSK9 inhibitors like PF-06446846 potentiates the effector function of CD8^+^ cells [85]. Together, these findings suggest a potential enhancement of immune therapy via promoting cholesterol synthesis and/or uptake in Teff cells and inhibiting cholesterol biosynthesis in Treg cells. Surprisingly, a previous paper has demonstrated that statins could also work as vaccine adjuvants given their role in inhibiting the geranylgeranylation of small GTPases, enhancing antigen presentation, and activating Th1 and cytolytic T cell responses [98]. However, it is important to note that the effect of statins is too upstream from cholesterol synthesis, and thus a cholesterol-independent effect may contribute to their antitumor effects, as well.

Modulating cholesterol efflux and esterification pathways are also potential strategies for treating cancers. LXR targets cancer cells, immune cells, and stromal cells, and plays anti-inflammatory functions. Therefore LXR serves as a promising target [99]. Inhibition of LXR using SR9243 shrinks tumors via promoting the cytotoxic activity and mitochondrial metabolism of CD8^+^ T cells in triple-negative breast cancer [100]. In another aspect, and consistent with the notion that LXR exerts both anti-inflammatory and pro-inflammatory functions, treatment with the LXR agonist RGX-104 robustly impedes tumor progression across a panel of malignancies. This drug effect is mediated by reduced MDSC abundance and increased cytotoxic T cell activation [55]. Therefore, the LXR activity requirement for different immune cells and cancer cells should be exploited in advance to achieve a better T cell response. In addition, blocking cholesterol esterification has been shown to increase plasma membrane cholesterol levels of CD8^+^ T cells, accompanied with efficient immunological synapse formation and enhanced T-cell receptor clustering and signaling. Consequently, drugs that target the key cholesterol esterification enzyme ACAT1, such as avasimibe, CP-113,818, and K-604, have been shown to augment CD8^+^ T cell functions and achieve a better antitumor outcome [57]. Importantly, combination of avasimibe with doxorubicin, paclitaxel, and an anti-PD-1-antibody exhibits better efficacy in treating different cancer models by invigorating CD8^+^ T cells [57, 101, 102]. Meanwhile, cholesterol sulfate or SR1078-mediated activation of retinoic acid-related orphan receptor α (RORα), a repressor for cholesterol esterification, also increases proliferation and IFN-γ production of CD8^+^ T cells in a colon cancer model [103]. Together, regulation of cholesterol esterification may contribute to stronger T cell effector responses and facilitate cancer treatment.

### Adoptive cell therapy

In addition to directly blocking T cells in the TME by metabolism-targeting drugs, adoptive cell therapy (ACT) also holds great potential to empower the immune system and attack tumors [104]. For instance, GW50156 (GW), an agonist of PPARα and PPARδ/β, is reported to potentiate the transcription of CPT1a and skews the bioenergetics profile of activated CD8^+^ T cells from glycolysis to FAO. GW50156-treated CD8^+^ T cells retain higher T-bet and IFNγ expression and demonstrate better persistence in the melanoma ACT model, therefore achieving a superior tumor inhibition capability [105]. Treating CD8^+^ T cells with another PPARα agonist, fenofibrate, also reprograms T cells to increased catabolism and lipid oxidation, which is instrumental for increasing T cell functionality and eliminating tumors in ACT [69]. Moreover, fenofibrate loaded on amphiphilic polygamma glutamic acid-based nanoparticles with anti-CD3 antibody modification (aCD3/F/AN) could be directly delivered into tumors through intratumoral administration, leading to an elevated activation of the FAO pathway and strengthening the tumor killing effect [106]. It is of interest that a recent study overexpressed FOXP3 in CD8^+^ T cells to gain a better antitumor activity [107]. In their findings, adoptive T cell therapy with FOXP3-overexpressing CD8^+^ T cells significantly restrains the melanoma tumor progression and prolongs mice survival. Further metabolic transcription analysis identified that FOXP3-overexpressing CD8^+^ TIL cells enrich the metabolic pathways like glycolysis, fatty acid metabolism, and OXPHOS, indicating that those FOXP3-engineered CD8^+^ T cells resemble the metabolic feature of tumor-infiltrating Treg cells and confer a survival advantage in a harsh TME [107]. However, one problem within this study is that FOXP3-expressing CD8^+^ T cells have impaired *in vitro* expansion capability. Moreover, a contrary study has shown that adoptive transfer of FOXP3 mutant CD8^+^ T cells exhibit better antitumor activity [108]. Therefore, a careful evaluation of the target genes of FOXP3 under different differentiation stages of T cells is warranted. Considering that oxidized lipids in the TME tend to cause peroxidation, overexpressing glutathione peroxidase 4 (GPX4), an antioxidant enzyme, in CD8^+^ T cells could rescue their functionality *in vivo* in the presence of oxidized low-density lipoproteins (OxLDL). Adoptive transfer of GPX4 overexpressing cells to mice also fulfills an effective antitumor function [64]. A similar conclusion is drawn by the use of ferroptosis inhibitors or inducers, as shown by the result that mice infused with ferroptosis inhibitor-treated CD8^+^ T cells demonstrate better efficacy in inhibiting tumor growth [65].

Engineered chimeric antigen receptor T (CAR-T) and TCR-T therapies that are based on cholesterol metabolism reprogramming are also applicable to treat cancers. For example, applying the RORγ agonist LYC-54143 during T-cell expansion enhances the efficacy of CAR-T cells with an elongated memory against tumors [109, 110]. In addition, CD3ζ/CD28/4-1BB mesothelin (MSLN)-targeting CAR-T cells carrying the ACAT1 inhibitor avasimibe or shRNA targeting ACAT1 show enhanced TCR clustering and increased cytotoxicity *in vivo*, yielding superior antitumor efficacy of ACAT1-engineered CAR-T cells in solid tumors [111]. Moreover, a study showing that clicking liposomal avasimibe onto the T cell surface by lipid insertion could sustain T cell activation by increasing the concentration of cholesterol in the T cell membranes. The adoptive transfer of cell-surface anchor-engineered T cells (T-Tre/bicyclo [6.1.0] nonyne (BCN)-Lipo-Ava cells) promotes the therapeutic efficacy in both a disseminated melanoma mouse model and an orthotopic glioblastoma mouse model [112]. Collectively, manipulating T cells according to their metabolic adaption is a promising approach to boost immunotherapy.

## Conclusion and perspective

In conclusion, distinct T cell subsets are characterized functionally by different lipid metabolic adaptions to execute their immune response, with fatty acid and cholesterol metabolism being the subjects of tremendous studies. Importantly, dedicated efforts have been made to understand the lipid metabolic fitness of T cells within the TME. These studies are of potential therapeutic importance and warrant further clinical investigation into reagents or adoptive cell therapies targeting lipid metabolism.

Despite the significant progress that has been made regarding lipid metabolism in T cells during the past years, several questions still need to be further elucidated. For example, molecular mechanisms underlying the disparate response in T cell subsets should be addressed in different disease contexts. To prevent cancer progression, Teff cells should prevail over Treg cells to execute immune surveillance roles, as to avoid autoimmune disease, and Treg cells are expected to suppress inflammatory events. Therefore, pinpointing the interplay and balance between inflammatory T cells and immunosuppressive T cells is of vital importance. Additionally, a subpopulation of TILs, named bystander CD8^+^ TILs, also exist in TME and recognize cancer-unrelated epitopes [113]. How these bystander T cells compete with functional T cells and cancer cells for lipid nutrition is of interest. A similar question can also be extended to other cells in the TME, like cancer cells and tumor-infiltrating immune cells, as different requirements for lipid metabolism have been demonstrated between cancer cells and immune cells. In this regard, though efforts have been devoted to targeting lipid metabolism for cancer therapy, no universal conclusions can be made regarding which of these strategies would be beneficial or deleterious, as the lipid metabolism regulation in the tissue milieu is quite complex and the clinical efficacy of any intervention may vary from patient to patient. Therefore, the fundamental mechanisms behind lipid metabolism may help to uncover previously unrecognized therapeutic vulnerabilities and overcome the side effects and limitations of current therapies.
